# Genomic Sequencing Profiles of *Mycobacterium tuberculosis* in Mandalay Region, Myanmar

**DOI:** 10.3390/tropicalmed8040239

**Published:** 2023-04-21

**Authors:** Aye Nyein Phyu, Si Thu Aung, Prasit Palittapongarnpim, Kyaw Ko Ko Htet, Surakameth Mahasirimongkol, Wuthiwat Ruangchai, Bharkbhoom Jaemsai, Htin Lin Aung, Htet Myat Win Maung, Angkana Chaiprasert, Petchawan Pungrassami, Virasakdi Chongsuvivatwong

**Affiliations:** 1 National Tuberculosis Programme, Department of Public Health, Ministry of Health, Mandalay 05071, Myanmar; 2 Department of Epidemiology, Faculty of Medicine, Prince of Songkla University, Hat Yai 90110, Thailand; 3 Department of Public Health, Ministry of Health, Keng Tung 06231, Myanmar; 4 Pornchai Matangkasombut Center for Microbial Genomics, Department of Microbiology, Faculty of Science, Mahidol University, Bangkok 10400, Thailand; 5 Medical Life Sciences Institute, Department of Medical Sciences, Ministry of Public Health, Nonthaburi 11000, Thailand; 6 Department of Microbiology and Immunology, University of Otago, Dunedin 9016, New Zealand; 7 Office of Research and Innovation, Faculty of Medicine Siriraj Hospital, Mahidol University, Bangkok 10700, Thailand; 8 Department of Disease Control, Ministry of Public Health, Nonthaburi 11000, Thailand

**Keywords:** genomic sequencing profiles, *Mycobacterium tuberculosis*, Mandalay region, Myanmar

## Abstract

This study aimed to characterize whole-genome sequencing (WGS) information of *Mycobacterium tuberculosis* (Mtb) in the Mandalay region of Myanmar. It was a cross-sectional study conducted with 151 Mtb isolates obtained from the fourth nationwide anti-tuberculosis (TB) drug-resistance survey. Frequency of lineages 1, 2, 3, and 4 were 55, 65, 9, and 22, respectively. The most common sublineage was L1.1.3.1 (n = 31). Respective multi-drug resistant tuberculosis (MDR-TB) frequencies were 1, 1, 0, and 0. Four clusters of 3 (L2), 2 (L4), 2 (L1), and 2 (L2) isolates defined by a 20-single-nucleotide variant (SNV) cutoff were detected. Simpson’s index for sublineages was 0.0709. Such high diversity suggests that the area probably had imported Mtb from many geographical sources. Relatively few genetic clusters and MDR-TB suggest there is a chance the future control will succeed if it is carried out properly.

## 1. Introduction

Although there are international efforts to fight tuberculosis (TB), it remains the main cause of death from a single pathogen [1]. Understanding the nature of the pathogen, *Mycobacterium tuberculosis* (Mtb), is crucial in the prevention and control program.

Whole-genome sequencing (WGS) is an effective way to disclose the nature of Mtb. Data from WGS can be used for the classification of Mtb into lineages and sublineages, with much better precision and accuracy than previous technologies such as restriction fragment length polymorphism (RFLP) [2]. With the evidence of certain genes associated with certain drug resistance, WGS can replace the conventional drug susceptibility test (DST) in the drug resistance survey. Moreover, the genetic distance between two or more isolates can be used to detect genetic cluster outbreaks of Mtb.

Worldwide, Mtb was classified into four major lineages (L), which are significantly linked with geography and host genetics [3]. Common areas of L1 to L4 are East Africa and Southeast Asia; Asia to Europe and Africa; South Asia, North Africa, and East Africa; and Europe and America [4,5,6,7].

Any areas with all sublineages in common would be considered as having high biodiversity, which may be due to the importing and maintenance of Mtb from various geographic sources. On the other hand, evidence of the genetic clusters would suggest the recent outbreak or the domination of transmission of a certain strain. 

In Myanmar, the estimated incidence of TB was 360 per 100,000 population. The estimated proportions of TB cases with multi-drug-resistant (MDR)/rifampicin-resistant tuberculosis (RR-TB) in 2021 were 4.1% (3.8–4.3) among new TB cases and 19% (18–20) among previously treated cases [1]. The Myanmar National TB Programme (NTP) conducts the anti-TB drug resistance survey every five years. The three previous surveys revealed the proportion of MDR-TB among new and previously treated TB patients to be 4.0% and 15.5% in 2002–2003; 4.2% and 10.0% in 2007–2008; and 5.0% and 27.1% in 2012–2013 [8]. The fourth nationwide anti-TB drug resistance survey was conducted in 2019–2020 [8]. Here, we report the Mandalay regional part of this fourth survey, which was the first survey using WGS. Although the study of Phyu et al. was the first to describe the distribution of lineages and drug resistances using WGS in Upper Myanmar [9], here we provide new information about genomic clusters, biodiversity, and sublineages, in detail, that were circulating in the Mandalay region. 

To broaden the understanding of Mtb genomics in Myanmar, readers are reminded that there was a similar study at Kayin State in the Myanmar Thailand Border area in 2019 [10]. The information contributed by our current report, when combined with that from the Kayin study, will provide valuable input for the management of the National TB Control Programme in Myanmar. 

The objectives of this study were (1) to construct the phylogenetic tree based on the genomic sequences of Mtb isolates obtained from the survey in the Mandalay region of Myanmar; (2) to classify the genotypes of isolates and examine the possible genetic clustering among the collected isolates in Mandalay region; (3) to compare the biodiversity and sublineages of TB isolates in Mandalay region and Kayin state in Myanmar; and (4) to describe the drug-resistance mutations with sublineages in Mandalay.

## 2. Materials and Methods

### 2.1. Study Sites

Mandalay is the major city in the middle part of Myanmar. The population of Mandalay region is around 6.2 million [11]. Notified MDR/RR-TB cases were 180 in 2020, the third rank in the whole country [12]. The Mandalay region consists of seven districts, which are subdivided into 28 townships. From the NTP survey design, our study sites covered five townships: Chanmyathazi, Kyaukpadaung, Meiktila, Sintgaing, and Singu townships. They are shown in Supplementary Figure S1.

Kayin state is situated in Lower Myanmar and adjacent to the Mandalay region. The population of Kayin state is 1.574 million [11]. It consists of five districts, which are subdivided into 7 townships. The study sites were three townships in Kayin state, and they were Hpa-An, Kawkareik, and Myawaddy [10].

### 2.2. Study Design

A cross-sectional study was carried out between February and August 2020 in the above five townships involving TB patients based on positive sputum culture regardless of their microscopic (for acid fast bacilli) and/or molecular study (Gene Xpert MTB/RIF Assay) results. The selected patients must not have been incarcerated at the time of selection and must never have received anti-TB treatment for more than seven days in their current regimen.

### 2.3. Participants

Of 28 townships of the Mandalay region, 5 were used because they could provide sputum samples from at least 10 smear positive consenting patients [8].

Morning sputum was collected on 3 consecutive days. The first specimen was sent for Gene Xpert test. The other two were sent for TB culture at the Upper Myanmar TB Laboratory if the first produced a positive Xpert test [8].

Based on the assumption that the proportion of MDR-TB was 0.05, the design effect was 1.2, and the 95% confidence limit was 0.04 from the estimate, the required sample size was 137 [13].

### 2.4. TB Culture Testing 

At the Upper Myanmar TB Laboratory with biosafety level 3, sputum samples were decontaminated and inoculated in Mycobacterium Growth Indicator Tube (MGIT) culture immediately on arrival, and within ≤3 days of sputum sample collection. For each sample, one Löwenstein–Jensen media (LJ) tube was inoculated in parallel to MGIT as a back-up, kept at 37 °C, and the sputum sample discarded thereafter, to ensure that re-culturing in MGIT was possible if the initial MGIT culture failed. For each specimen, and for each positive MGIT culture that was confirmed as Mtb using the Capilia TB Immunochromatographic test (ICT) kit, at least one aliquot of viable culture was cryopreserved in glycerol at −80 °C, and the remaining culture tube was sub-cultured onto 2 LJ tubes. One positive sub-cultured LJ tube was used for DNA extraction for WGS, and the other tube was stored at −80 °C as a back-up, in case further DNA extractions were required [8].

Among the 221 smear positive or Xpert MTB/RIF positive samples, 206 samples were successfully cultured. 

### 2.5. Extraction of Genomic DNA

At the Upper Myanmar TB Laboratory, genomic DNA was extracted from sputum culture isolates. MoBio Microbial DNA Isolation Kits, (Qigen DNEasy Ultraclean Microbial DNA extraction kit, Cat No: 12224-50) was used [14,15,16,17]. Both first- and second-line line probe assay (LPA) were tested by the MTB DRplus kit and the MTB DRsl kit. The extracted DNA was kept at −80 °C and sent to University of Otago, New Zealand via the National TB Reference Laboratory every 3 months for WGS or as necessary according to the international regulation to assure the stability of extracted DNA. 

### 2.6. Sequencing at University of Otago

At University of Otago, extracted DNA was sequenced using Illumina MiSeq (https://www.illumina.com, accessed on 5 February 2021) as previously described [15,16]. The sequencing data (FASTQ) were stored on the University of Otago’s high-capacity central file storage (HCS) server.

### 2.7. Data Analysis Steps

#### 2.7.1. Genomic Characterization

At the Faculty of Science, Mahidol University, Bangkok, Thailand, we used the snpplet pipeline for processing short-read sequencing data to obtain short variants (SNPs and indels). Using trimmomatic v0.39, the short reads were trimmed to remove adapter sequences and low-quality read positions (sliding-window trimming with a window size of 4 and a read quality threshold of 30) [18]. The trimmed reads were then mapped to the H37Rv reference genome (NC_000962.3) using bwa mem [19]. Picard’s MarkDuplicates was used to identify duplicate reads before a per-sample variant calling using GATK (genome analysis toolkit) HaplotypeCaller in a haploid model [20], excluding bases with a quality score below 20. To compare SNPs across samples, we performed joint genotyping of all samples using GATK GenotypeGVCFs [20], using per-sample variant calls as inputs. We used mtbtyper for genotyping Mtb isolates from WGS data **(**https://github.com/ythaworn/mtbtyper, accessed on 15 October 2022).

#### 2.7.2. Phylogenetic Analysis

**Figure 1:**
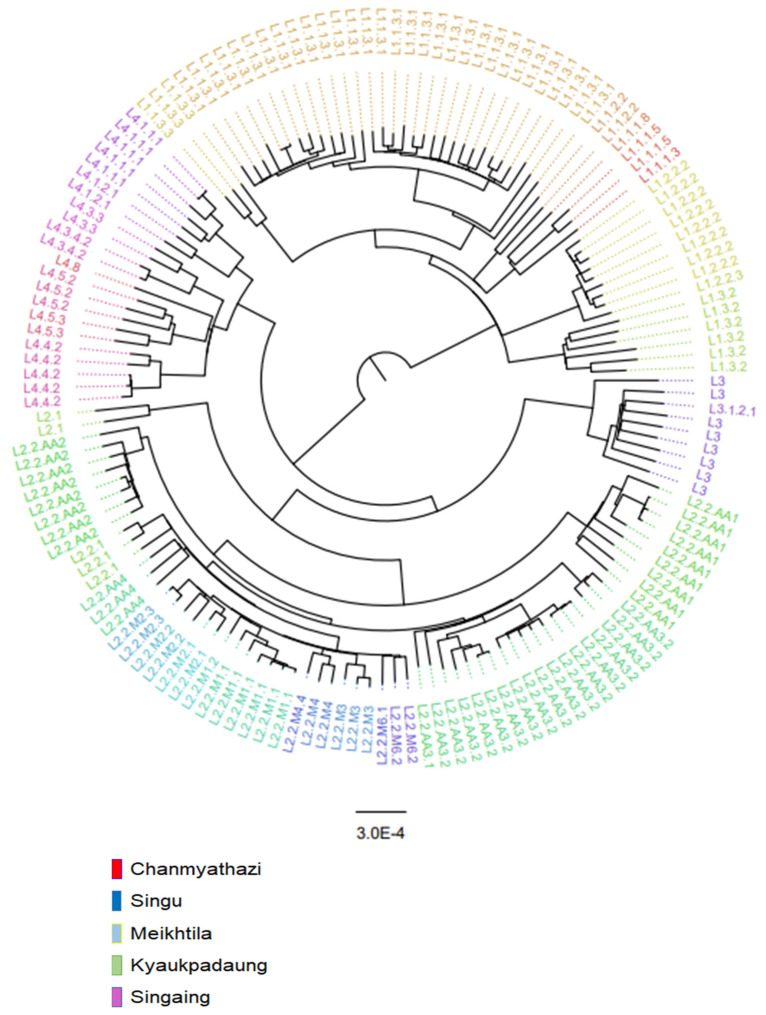
A maximum-likelihood phylogenetic tree of 151 *Mycobacterium tuberculosis* isolates from five townships in the Mandalay region, Myanmar.

A maximum-likelihood (ML) phylogenetic tree of 151 isolates was inferred using IQ-TREE v2 [21] with ultrafast bootstrap supports from 1000 replications (Figure 1). For the quality of WGS, this study cut off the coverage and read depth of sequencing at 90 and 15, respectively. The phylogenetic tree was constructed from 151 isolates. The phylogenetic tree was visualized using the FigTree program version 1.4.4 (http://tree.bio.ed.ac.uk/software/figtree/, accessed on 20 October 2022). The best-fit nucleotide substitution model was K3Pu + F+ASC + R4 chosen according to the Bayesian Information Criterion (BIC) as determined by ModelFinder [22]. The lineage 4 H37Rv reference strain (Gene Bank accession number NC_000962.3) was used as an outgroup for rooting the tree.

#### 2.7.3. Identification of Genetic Clusters

Pairwise SNV distances were calculated using Molecular Evolutionary Genetics Analysis Version 11 (MEGA11) (https://doi.org/10.1093/molbev/msab120, accessed on 20 October 2022) [23]. We identified genomic clusters as clades in the phylogeny containing isolates that can be linked via pairwise single nucleotide polymorphism (SNP) distances. If the pairwise distance between two isolates was <20 SNPs, they were considered closely related or genomically linked [24]. A cluster contains an aggregate of pairs of isolates in which each one differs by fewer than 20 SNPs from at least one of the other elements of the cluster. 

#### 2.7.4. Biodiversity

At the Department of Epidemiology, Faculty of Medicine, Prince of Songkla University, Hat Yai, Thailand, Simpson’s index was calculated based on the formula:

D=Ʃn(n−1)/N(N−1),

where *D* is Simpson’s index, *n* is the total number of organisms of a particular species, *N* is the total number of organisms of all species, and Ʃ means “sum up”. The scale ranges from 0–1, with 0 representing the highest biodiversity of a particular species and 1 representing the lowest biodiversity of a particular species [25,26].

#### 2.7.5. Drug Resistance

We used TB-profiler software for the prediction of drug-resistance mutation [27].

#### 2.7.6. Merging with Kayin State Data

The 109 samples of sequencing data for Kayin state were downloaded from the European Nucleotide Archive (ENA) of EMBL-EBI mirrored in the Sequence Read Archive (SRA) database [10].

### 2.8. Statistical Analysis

Statistical analyses were performed using R version 4.0.2 (R foundation for Statistical Computing, Vienna, Austria). The Mtb major lineages, sublineages, and their drug-resistance mutations were presented as frequency and percentage. Information on drug resistance was limited to only untreated cases in order to assess only primary drug resistance [28], which better reflected the severity of the problem. The relationship between the geographical areas of Mandalay and Mtb lineages, and between anti-TB drug resistance and major lineages, was analyzed using the Chi-squared test. The significance level was set at a *p* value of less than 0.05.

## 3. Results

### 3.1. Participant Characteristics

The median age of the participants was 42 (IQR: 31.5–54.0) years. Males accounted for 70%. There were no significant association between Mtb major lineages and the age and gender of the participants; or townships (Table 1).

### 3.2. Phylogenetic Analysis

A maximum-likelihood phylogenetic tree of 151 *Mycobacterium tuberculosis* isolates from five townships is shown in Figure 1, with the names of sublineages labeled in color at the outermost part. The majority of isolates belonged to lineage 1 (L1) and L2. Intriguingly, among L1, the majority of isolates belonged to L1.1.3 (62%, 34/55), which belongs to the spoligogroup EAI6_BGD. L1.1.3.1 is the predominant subgroup of L1.1.3 (91%, 31/34), similar to the Kayin state. L1.1.2.2 and L1.3, predominant sublineages in India, were also identified, albeit in lower proportions than in the Kayin state. Among the L2.2 (Beijing, China) isolates, the majority (65%, 41/63) belonged to the ancestral group, with L2.2.AA3.2 being the highest. There were no predominant modern Beijing sublineage in the Mandalay region. L4.4. and L4.5, which are predominant L4 strains in China, constitute 45% (10/22) of the L4 strains.

### 3.3. Identification of Genetic Clusters

There were four clusters identified based on the pairwise distance. The number of isolates in the respective lineages were 3 (L2), 2 (L4), 2 (L1), and 2 (L2). 

### 3.4. Distribution of Sublineages and Biodiversity in Mandalay Region and Kayin State 

The distribution of sublineages and the biodiversity in Mandalay region and Kayin state are shown in Table 2. The overall Simpson’s indices were 0.0709 in Mandalay and 0.072885 in Kayin. Sublineage L1.1.3.1 was the most common (n = 31), followed by L2.2.AA3.2 (n = 17), which was available only in Mandalay. In Kayin, the most prominent sublineages were L1.1.3.1 (n = 17) and L1.3 (n = 17). The latter was only in Kayin. 

### 3.5. Distribution of Drug-Resistance Mutation and Sublineages in Mandalay Region

The distribution of drug-resistance mutation and sublineages is shown in Table 3. Among the isoniazid-resistant strains, 70% were S315T mutations in the *katG* gene. Regarding the distribution of streptomycin drug resistant mutation, there were 73% resistant isolates with an *rpsL* K43R mutation, which was the most common occurrence in the Mandalay region.

Among 126 new TB patients, MDR-TB accounted for 1.6% (2/126) and poly-drug resistance 5.6% (7/126). MDR-TB (isoniazid and rifampicin resistance) genes were found in L1 and L2. Poly-drug resistance was also found in L1 (n = 2), L2 (n = 4), and L4 (n = 1). There was no drug resistance identified in seven isolates of L3.

## 4. Discussion

We identified L1, L2, L3, and L4 in our samples. There were no associations between lineages and demographic variables. The most common sublineage was L1.1.3.1 (n = 31), followed by L2.2.AA3.2 (n = 17), which was exclusively found in Mandalay. In Kayin, the most prevalent sublineages were L1.1.3.1 (n = 17) and L1.3 (n = 17), with the latter being unique to Kayin. There were four small clusters, and the level of biodiversity was high. Streptomycin and isoniazid were the two most commonly observed drug-resistance mutations. 

Four types of major lineages and 35 sub-lineages were identified among 151 isolates via WGS in our study. Lineage 1 is divided into five major sub-lineages, i.e., L1.1.1, L1.1.2, L1.1.3, L1.2, and L1.3. L1.1 is made up of three additional sublineages, L1.1.1–1.1.3 [3]. Specifically, L1.2.1.1 is found in Southern Taiwan and Indonesia, accounting for >90% and roughly 40% of EAI isolates, respectively [29]. In the Philippines, 80% of isolates are L1.2.2 [30]. China has mostly Modern Beijing, L4.4 and L4.5 [31].

Many studies have suggested that the L1.1.3 sublineage is more virulent and more resistant to anti-TB drugs. According to the research done in South Africa, active TB cases infected with L1.1.3 strains were at a higher risk of developing drug-resistant strains and treatment failure than those infected with other strains [32]. Another study conducted in Ethiopia found that the L1.1.3 sublineage was associated with a higher risk of treatment failure and death among TB patients, compared to other Mtb strains [33]. The prominence of the L2.2.AA3.2 sublineage in Mandalay is not fully understood.

Our current study revealed relatively few outbreaks of TB genetic clusters. These findings imply the simultaneous transmission of different lineages imported from different sources. Simpson’s index (D) was 0.0709 in our study and 0.07 reported by Maung et al. in Kayin state [10], much lower than that of 0.68 in the Philippines [30] and 0.21 in China [31]. It has been proposed that the primary causes of genetic diversity within the *Mycobacterium tuberculosis* complex (Mtbc) may be attributed to the relative fitness and adaptability of imported genotypes to ecological and other unknown environmental factors [34]. 

The two most common drug-resistance mutations found were streptomycin (n = 11, 7%) and isoniazid (n = 10, 7%), which have been extensively used in the past. The first Mtb drug resistance to be identified was to streptomycin [35]. Of the streptomycin-resistant isolates, 73% had a *rpsL* K43R mutation, which confers high-level resistance to streptomycin by reducing the binding affinity of the drug to the ribosome [36]. This was the most common occurrence in the Mandalay region. A previous study from Upper Myanmar also reported resistant streptomycin 32 isolates with a *rpsL* K43R mutation [9]. In Asia, *rpsl* mutations were prevalent [37,38,39,40]. In Myanmar, streptomycin is utilized in developing specialized treatment for hepatotoxic TB patients who are not suitable for the standard anti-TB treatment regimen [9]. S315T mutations in the *katG* gene accounted for 70% of the isoniazid-resistant strains in our study. There were 22 resistant isoniazid isolates with the *katG* S315T mutation in a previous study from Upper Myanmar [9]. The *katG* gene encodes for the enzyme catalase-peroxidase, which is involved in the activation of isoniazid, a major component of anti-TB treatment [41]. A significant mechanism for isoniazid resistance in Mtb is via mutations in the *katG* gene [41]. 

Our study only obtained isolates from culture-positive TB patients. The findings cannot be generalized to smear negative and extra-pulmonary TB patients. We also did not have metadata on ethnicity, socio-economic status, traveling history, and treatment compliance. These limitations should be taken into account for TB control planning.

## 5. Conclusions

The current data suggest that the area probably had imported Mtb from many geographical sources. With relatively few genetic clusters and MDR-TB, there is a good chance that the future control will succeed if it is carried out properly.

## Figures and Tables

**Table 1:** Frequency (%) of major lineages of *Mycobacterium tuberculosis* by demographic variables.

	Lineage 1 N = 55 (%)	Lineage 2 N = 65 (%)	Lineage 3 N = 9 (%)	Lineage 4 N = 22 (%)	*p* Value *
Age in year					0.903
<20	4 (7.3)	4 (6.2)	0 (0.0)	1 (4.5)	
20–30	8 (14.5)	15 (23.1)	0 (0.0)	5 (22.7)	
31–40	15 (27.3)	13 (20.0)	2 (22.2)	4 (18.2)	
41–50	12 (21.8)	16 (24.6)	3 (33.3)	5 (22.7)	
51–60	7 (12.7)	10 (15.4)	3 (33.3)	3 (13.6)	
>60	9 (16.4)	7 (10.8)	1 (11.1)	4 (18.2)	
Gender					0.066
Male	34 (61.8)	44 (67.7)	9 (100)	18 (81.8)	
Female	21 (38.2)	21 (32.3)	0 (0)	4 (18.2)	
Township					0.304
Chanmyathazi	8 (14.5)	11 (16.9)	1 (11.1)	4 (18.1)	
Kyaukpadaung	8 (14.5)	13 (20.0)	5 (55.6)	5 (22.8)	
Meiktila	14 (25.5)	12 (18.5)	1 (11.1)	2 (9.1)	
Singaing	7 (12.7)	6 (9.2)	0 (0.0)	2 (9.1)	
Singu	18 (32.8)	23 (35.4)	2 (22.2)	9 (40.9)	

* *p* value was calculated from Chi-squared test (X^2^).

**Table 2:** Distribution of sublineages and biodiversity in Mandalay region and Kayin state.

Sublineages	Mandalay Region (n)	Kayin State (n)
L1.1.1	0	8
L1.1.1.2	0	1
L1.1.1.3	1	0
L1.1.1.5	2	2
L1.1.1.7	0	1
L1.1.1.8	1	3
L1.1.2.1	0	1
L1.1.2.2 (EAI3_IND)	2	10
L1.1.3	0	3
L1.1.3.1 (EAI6_BGD)	31	17
L1.1.3.3 (EAI6_BGD)	3	0
L1.2.2	0	1
L1.2.2.1	0	2
L1.2.2.2	0	6
L1.2.2.3	0	1
L1.3	0	17
L1.2.2.2 (EAI2_NTB)	8	0
L1.2.2.3	1	0
L1.3.2	6	0
L2.1 (ProtoBeijing)	2	0
L2.2 (Unclassified Ancestral)	3	0
L2.2.AA.1	9	1
L2.2.AA.2	8	1
L2.2.AA.3	0	1
L2.2.AA3.1	1	0
L2.2.AA3.2	17	0
L2.2.AA4	3	3
L2.2.M1.1 (Pacific RD150)	6	2
L2.2.M1.2	1	0
L2.2.M2	0	8
L2.2.M2.1 (Asian African 2)	2	0
L2.2.M2.2 (Asian African 2)	2	0
L2.2.M2.3	2	0
L2.2.M3 (Asian African 3)	3	1
L2.2.M4	2	0
L2.2.M4.1 (Bmyc22+)	0	1
L2.2.1(Modern)	0	8
L2.2.M4.4	1	0
L2.2.M6.1	1	0
L2.2.M6.2 (Asian African 1)	2	0
L3	8	2
L3.1.2	0	1
L3.1.2.1	1	1
L4.1.1.1	5	0
L4.1.2	0	1
L4.1.2.1	2	0
L4.3	0	1
L4.3.3	2	0
L4.3.4.2	2	0
L4.4	0	1
L4.4.2	5	0
L4.5	0	1
L4.5.2	5	0
L4.5.3	0	1
L4.8	1	1
Biodiversity Index (D)	0.0709	0.072885

**Table 3:** Frequency distribution of drug-resistance mutation and sub-lineages in Mandalay region.

Drug-Resistance Mutation	N = 151	Sub-Lineage
Isoniazid		
*InhA*_C15T	2	L2.2.AA2, L4.3.3
*katG*_S315T	7	L2.2.AA1 (1), L1.1.3.1 (3), L2.2.AA3.2 (2), L1.2.2.2 (1)
*inhA*_p.Ile21Val	1	L2.2.M2.1
Rifampicin		
*rpoB*_S450L	2	L1.1.3.1
*rpoB*_S450L	1	L2.2.AA3.2
Pyrazinamide		
*ncA*_408_ins_1_a_at	1	L1.1.3.1
Ethambutol		
*embB*_M306V	1	L1.1.3.1
*embC*_c-516t	1	L1.2.2.2
Streptomycin		
*rpsL*_K43R	8	L2.2.AA1 (1), L4.4.2 (1), L4.5.3 (1), L1.1.3.1 (2), L2.2.AA3.2 (2), L2.2.M2.1 (1)
*rpsL*_K88R	1	L1.1.3.1
*gidB*_A138V	1	L1.1.3.1
Other (rpsL_p.Lys43Arg)	1	L2.2.AA3.2
Levofloxacin		
*gyrA*_D94G	1	L1.1.3.1
*gyrA*_A90V	2	L1.1.3.1 (2), L2.2.AA3.2 (1)
Ethionamide		
*inhA*_C15T	1	L4.3.3

## Data Availability

All of the analyzed data are included in this published article.

## Supplementary Materials

The following supporting information can be downloaded at: https://www.mdpi.com/article/10.3390/tropicalmed8040239/s1, Figure S1: Selected five townships in Mandalay region.
