# Supplementary material for: Genomic Sequencing Profiles of Mycobacterium tuberculosis in Mandalay Region, Myanmar
Source: Trop Med Infect Dis. 2023 Apr 21;8(4):239. doi: 10.3390/tropicalmed8040239 (PMC10141229; doi:10.3390/tropicalmed8040239)
Supplement: Supplementary file 1 [file tropicalmed-08-00239-s001.zip › tropicalmed-2285468-supplementary.pdf]

Supplementary File

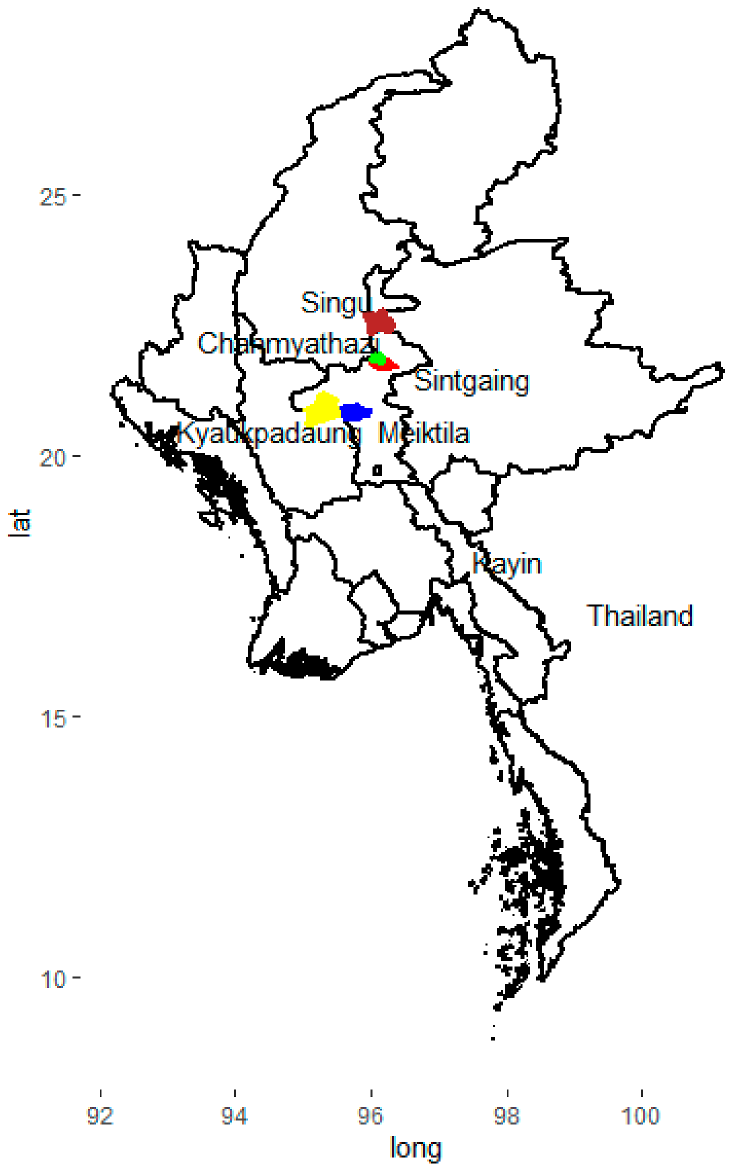

Figure S1. Selected five townships in Mandalay region.

The selected five townships in Mandalay region was shown in Figure S1. The brown color indicates the Singu township, the green color the Chanmyathazi township, the red color the Sintgaing township, the yellow color the Kyaukpadaung township and the blue color the Meiktila township.
